# Theranostic Microbubbles with Homogeneous Ligand Distribution for Higher Binding Efficacy

**DOI:** 10.3390/pharmaceutics14020311

**Published:** 2022-01-28

**Authors:** Simone A. G. Langeveld, Bram Meijlink, Inés Beekers, Mark Olthof, Antonius F. W. van der Steen, Nico de Jong, Klazina Kooiman

**Affiliations:** 1 Thorax Center, Biomedical Engineering, Erasmus University Medical Center Rotterdam, 3000 CA Rotterdam, The Netherlands; k.meijlink@erasmusmc.nl (B.M.); d.beekers@erasmusmc.nl (I.B.); m.olthof@erasmusmc.nl (M.O.); a.vandersteen@erasmusmc.nl (A.F.W.v.d.S.); n.dejong@erasmusmc.nl (N.d.J.); k.kooiman@erasmusmc.nl (K.K.); 2 Department of Health, ORTEC B.V., 2719 EA Zoetermeer, The Netherlands; 3 Imaging Physics, Delft University of Technology, 2628 CJ Delft, The Netherlands

**Keywords:** phospholipid-coated microbubbles, binding efficacy, ligand distribution, ultrasound molecular imaging, targeted drug delivery

## Abstract

Phospholipid-coated targeted microbubbles are used for ultrasound molecular imaging and locally enhanced drug delivery, with the binding efficacy being an important trait. The use of organic solvent in microbubble production makes the difference between a heterogeneous or homogeneous ligand distribution. This study demonstrates the effect of ligand distribution on the binding efficacy of phospholipid-coated α_ν_β_3_-targeted microbubbles in vitro using a monolayer of human umbilical-vein endothelial cells and in vivo using chicken embryos. Microbubbles with a homogeneous ligand distribution had a higher binding efficacy than those with a heterogeneous ligand distribution both in vitro and in vivo. In vitro, 1.55× more microbubbles with a homogeneous ligand distribution bound under static conditions, while this was 1.49× more under flow with 1.25 dyn/cm^2^, 1.56× more under flow with 2.22 dyn/cm^2^, and 1.25× more in vivo. The in vitro dissociation rate of bound microbubbles with homogeneous ligand distribution was lower at low shear stresses (1–5 dyn/cm^2^). The internalized depth of bound microbubbles was influenced by microbubble size, not by ligand distribution. In conclusion, for optimal binding the use of organic solvent in targeted microbubble production is preferable over directly dispersing phospholipids in aqueous medium.

## 1. Introduction

Microbubbles are a type of ultrasound contrast agents that consist of a gas core with phospholipid, polymer or protein coating [1]. With a diameter of 1 to 10 µm, microbubbles are confined to the vasculature and therefore function as a blood pool marker in diagnostic ultrasound imaging [2]. The gas core of a microbubble responds to an ultrasound wave by compressing and expanding, referred to as oscillation. Depending on the ultrasound pressure and frequency, this acoustic response results in stable or inertial cavitation and elicits a nonlinear signal that provides contrast to the surrounding tissue [3]. Microbubble oscillation can induce several biological effects, such as sonoporation—i.e., pore formation [4]—opening of cell–cell junctions [5], and stimulated endocytosis [6]. Together these effects result in locally enhanced drug uptake, which is particularly useful for targeted delivery of chemotherapeutics [7,8,9] and for reversible opening of the blood–brain barrier [10,11].

Phospholipid-coated microbubbles have a thinner shell than polymer- or protein-coated microbubbles, making them less stiff and more echogenic [12,13,14]. Furthermore, phospholipid-coated microbubbles can be functionalized with the addition of a ligand targeted to a specific biomarker [15,16]. Targeted microbubbles can be used for ultrasound molecular imaging [17,18,19], and when combined with therapeutic agents they function as theranostic agents [20,21,22]. A recent review on targeted microbubbles stated that when comparing the therapeutic effects of targeted and non-targeted microbubbles, treatment with targeted microbubbles always resulted in a significantly better therapeutic outcome [23]. Clinical translation is ongoing, with one microbubble formulation targeted to vascular endothelial growth factor receptor 2 (VEGFR2) showing potential for diagnostic imaging in patients with prostate cancer [24] and breast and ovarian lesions [19], both first-in-human studies. Besides clinical use, targeted microbubbles are already being applied in pre-clinical studies as ultrasound molecular imaging agents for studying disease progression [25,26] and monitoring response to therapy [27,28,29].

While it is clear that targeted microbubbles are a powerful diagnostic and therapeutic tool, translation from the pre-clinical phase to clinical studies requires extensive safety studies. Furthermore, pre-clinical drug development studies involving targeted microbubbles need standardization of microbubble production methods and ultrasound molecular imaging protocols [30]. An abstract first published by Klibanov et al. [16] is often referred to in papers where phospholipid-coated targeted microbubbles were produced by directly dispersing the coating components in aqueous medium [15,25,31,32,33] such as phosphate buffered saline (PBS), i.e., the direct method [34]. Others reported dissolving the coating components first in organic solvent, then drying to form a lipid film and finally dispersing the lipid film in aqueous medium [35,36,37,38], i.e., thin-film hydration or the indirect method [34]. Both production methods have been used for in vitro [16,34,35,36,37] as well as in vivo [15,25,31,32,33,38] studies. Our previous study comparing the spatial ligand distribution over the surface of 1,2-distearoyl-*sn*-glycero-3-phosphocholine (DSPC)-based microbubbles functionalized by streptavidin–biotin coupling showed that microbubbles produced by the direct method had a heterogeneous ligand distribution, in contrast to the microbubbles produced by an indirect method, which had a homogeneous ligand distribution [34]. However, to the best of our knowledge no studies have been done to directly compare the binding efficacy of microbubbles with the same coating composition with either a homogeneous or heterogeneous ligand distribution. Improved binding efficacy entails higher numbers of bound microbubbles, which is expected to improve the ultrasound molecular imaging signal [39] and, for therapeutic applications, has been shown to increase drug uptake [40].

The aim of this study was to determine if a homogeneous ligand distribution in targeted microbubbles results in different binding efficacy than a heterogeneous ligand distribution. Microbubbles produced with a direct method, resulting in heterogeneous ligand distribution, were compared to microbubbles produced with an indirect method, resulting in homogeneous ligand distribution. The binding efficacy was determined in vitro by confocal microscopy imaging of α_ν_β_3_-targeted microbubbles bound to human umbilical-vein endothelial cells (HUVECs) cultured under static conditions and under physiologically relevant flow. The chorioallantoic membrane (CAM) vessels of chicken embryos were used to evaluate the binding efficacy of α_ν_β_3_-targeted microbubbles in vivo. A recent study showed that the microbubble–cell interaction upon binding led to microbubble internalization, which affected the drug delivery outcome after ultrasound insonification (REF Beekers et al. “The 3D microbubble-cell dynamics: microbubble internalization and drug delivery by pores and tunnels”, under review). Hence, the 3D morphology of direct and indirect α_ν_β_3_-targeted microbubbles bound to HUVECs under static conditions was investigated, to determine the effect of ligand distribution on the internalization of α_ν_β_3_-targeted microbubbles.

## 2. Materials and Methods

### 2.1. Targeted Microbubble Preparation

Phospholipid-coated microbubbles were produced with a C_4_F_10_ gas core as described previously [34,39]. Two methods were used, and the resulting microbubbles will be referred to as direct and indirect microbubbles. For both methods the coating consisted of 84.8 mol% DSPC (Lipoid GmbH, Ludwigshafen, Germany), 8.2 mol% PEG40-stearate (Sigma-Aldrich, Zwijndrecht, The Netherlands), 5.9 mol% DSPE-PEG2000 (Iris Biotech GmbH, Marktredwitz, Germany), and 1.1 mol% DSPE-PEG2000-biotin (Avanti Polar Lipids, Alabaster, AL, USA). For the indirect method, all coating components were first dissolved in chloroform/methanol (9:1 vol/vol), the solvent was then evaporated using argon gas (Linde Gas Benelux, Schiedam, The Netherlands) and the lipids were freeze-dried (Alpha 1–2 LD plus, Martin Christ GmbH, Osterode am Harz, Germany) for 2 h. The obtained lipid film was rehydrated by adding PBS saturated with C_4_F_10_ gas (F2 Chemicals, Preston, UK), fluorescently labeled by adding lipid dye DiD (1,1′-dioctadecyl-3,3,3′,3′-tetramethylindodicarbocyanine perchlorate, Thermo Fisher Scientific, Waltham, MA, USA), placed in a sonicator bath for 10 min and subsequent probe sonication with a Sonicator ultrasonic processor XL2020 (HeatSystems, Farmingdale, NY, USA) at power setting 3 for 5 min (20 kHz). For the direct method, all coating components were dispersed directly in PBS saturated with C_4_F_10_, mixed, and lipid dye DiI (1,1′-dioctadecyl-3,3,3′,3′-tetramethylindocarbocyanine perchlorate, Thermo Fisher Scientific, Waltham, MA, USA) was added for fluorescent labeling of the direct microbubbles. For both methods the final concentration was 2.5 mg/mL DSPC, 0.625 mg/mL PEG40-stearate, 0.625 mg/mL DSPE-PEG2000 and 0.125 mg/mL DSPE-PEG2000-biotin. Microbubbles were then produced by sonication at 20 kHz (power setting 10) at the gas/water interface for 1 min under a constant stream of C_4_F_10_ gas.

After production, microbubbles were functionalized to target the α_ν_β_3_ integrin by biotin–streptavidin coupling, as described previously [15,41]. In short, microbubbles were first washed three times by centrifugation for 1 min at 400× *g* using PBS saturated with C_4_F_10_. The microbubble concentration was determined using a Coulter Counter Multisizer 3 (50 µm aperture tube, Beckman Coulter, Mijdrecht, The Netherlands) and 6 × 10^8^ microbubbles were incubated with 60 µg of streptavidin (2 mg/mL in PBS, Sigma-Aldrich, Zwijndrecht, The Netherlands) for 30 min on ice, followed by washing by centrifugation. Next, the streptavidin-conjugated microbubbles were incubated with 6 µg biotinylated antihuman CD51/61 antibody (i.e., anti- α_ν_β_3_, 304412, BioLegend, San Diego, CA, USA) for 30 min on ice. After incubation the α_ν_β_3_-targeted microbubbles were washed again, the concentration and size distribution were determined by Coulter Counter, and a mixed sample with a 1:1 ratio of indirect:direct was prepared, from here on referred to as stock sample. A new sample of microbubbles was functionalized for each experimental day, six batches of microbubbles were produced in total, and samples were taken from a batch of microbubbles up to 21 days post-production.

### 2.2. Endothelial Cell Culture

All in vitro experiments were performed using primary human umbilical-vein endothelial cells (HUVECs; C2519A, Lonza, Verviers, Belgium) from pooled donors, cultured with Endothelial Cell Growth Medium (EGM)-2 (Lonza) and grown to full confluency at 37 °C with 5% CO_2_ in T75 flasks in a humidified incubator. HUVECs were detached before each experiment with Accutase solution (Sigma-Aldrich, Zwijndrecht, The Netherlands). HUVECs used for experiments were between passage number 4 and 9. For experiments in static conditions, HUVECs were replated onto the bottom membrane of a CLINIcell (Mabio, Tourcoing, France) with 50 µm membranes (25 cm^2^) in 12 mL EGM-2, and incubated for 2 days at 37 °C with 5% CO_2_ to achieve a confluent monolayer. In total, eight CLINIcells were used for binding efficacy experiments and four CLINIcells were used for internalization experiments. For experiments under flow, IbiTreat polymer µ-slides (80196, 0.8 mm channel height, Ibidi GmbH, Gräfelfing, Germany) were first pre-coated by incubating with 7.6 µg fibronectin (Roche, Basel, Switzerland) in 200 µL PBS at 37 °C with 5% CO_2_ for 1 to 2 h as instructed by Ibidi, then washed with 800 µL PBS. After seeding in the µ-slide, the HUVECs were placed in the humidified incubator at 37 °C with 5% CO_2_ without flow for 2 h to attach, then connected to an Ibidi fluidic unit and the computer-controlled Ibidi Pump system (Ibidi) with corresponding perfusion sets (Perfusion set Yellow and Green, 10964, Ibidi). For later injection of fluorescent dyes and microbubbles, two Luer injection ports (Ibidi) were inserted in the perfusion set: one at 3.5 cm downstream from the µ-slide and the other 7 cm upstream from the µ-slide. Laminar flow was started with controlled shear stress at 2.5 dyn/cm^2^ for 30 min, then 5.0 dyn/cm^2^ for 30 min, and finally 7.5 dyn/cm^2^ until at least 48 h post-seeding to obtain a confluent monolayer. In total six µ-slides were used to evaluate binding efficacy under flow, and eight µ-slides were used to evaluate dissociation of bound microbubbles under increasing flow.

### 2.3. CAM Model Preparation

For in vivo experiments, the CAM was prepared as described previously [42]. All animal experiments were conducted in accordance with The Netherlands Experiments on Animals Act and in accordance with the European Council (2010/63/EU) on the protection of animal use for scientific purposes. In short, freshly fertilized eggs (Drost Pluimveebedrijf Loenen BV, Loenen aan de Vecht, The Netherlands) were incubated for 5 days at 37 °C in a humidified incubator. The chicken embryo was taken out of the eggshell and placed into a weighing boat with the embryo and CAM on top. First a mixture of 2 µL Hoechst 33342 (10 mg/mL, Thermo Fisher Scientific, Waltham, MA, USA) to stain the cell nuclei, 3 µL CellMask™ Green Plasma Membrane Stain (5 mg/mL, Thermo Fisher Scientific, Waltham, MA, USA) to stain the ce ll membrane, and 5 µL targeted microbubbles (indirect and direct 1:1 ratio) was prepared, then 5 µL of this mixture was injected into a CAM vein using a capillary glass needle with a VisualSonics micro injection system (Fujifilm VisualSonics, Toronto, ON, Canada). A CLINIcell (Mabio, Tourcoing, France) was prepared by cutting out the top membrane and filling the frame with a solution of 2% agarose (A9539, Sigma-Aldrich, Zwijndrecht, The Netherlands) in demi water. The membrane containing the chicken embryo and the CAM was cut out, rinsed with PBS, and pinned down on the prepared CLINIcell for imaging with a confocal microscope. The heart rate was monitored prior to and during the imaging and a total of seven chicken embryos were used for in vivo experiments.

### 2.4. Confocal Microscopy Imaging

For the in vitro binding efficacy and dissociation and in vivo binding efficacy experiments, fluorescent images were acquired with a custom-built Nikon A1R+ confocal microscope [43] equipped with a 60× water dipping objective (NIR Apo 1.0W DIC, Nikon Instruments, Amsterdam, The Netherlands). For the in vitro 3D imaging of microbubble internalization, the microscope was equipped with a 100× water dipping objective (CFI Plan 100XC W, Nikon Instruments). Four channels were used to capture the different fluorescent signals: Hoechst was excited at 405 nm and detected at 450/50 nm (center wavelength/bandwidth), CellMask Green was excited at 488 nm and detected at 525/50 nm, DiI was excited at 561 nm and detected at 595/50, and DiD was excited at 640 nm and detected at 700/75 nm. All imaging experiments were performed in a custom-made water bath at 37 °C positioned beneath the microscope and completed within 2 h after placing the sample in the confocal set-up.

### 2.5. Binding Efficacy In Vitro Static

Statically cultured HUVECs in the CLINIcells were first incubated with CellMask™ Green Plasma Membrane Stain (4 µg/mL final concentration) for 10 min inside the humidified incubator to stain the cell membranes. Then, to stain the cell nuclei, Hoechst 33342 (5 µg/mL final concentration) was added together with the microbubbles (direct/indirect 1:1 ratio, 1 × 10^6^ microbubbles/mL final concentration) and the CLINIcell was placed with the HUVEC monolayer on top in the incubator for 5 min, to allow the targeted microbubbles to float up and bind to the cells. The CLINIcell top membrane was cut out to image the cells with an objective with a smaller working distance than the 5 mm between the CLINIcell top and bottom membranes [5] and the CLINIcell was then placed in the confocal set-up in a water bath at 37 °C with the HUVEC monolayer on the bottom membrane. Each confocal microscopy field of view (FOV) covered 210 × 210 µm (512 × 512 pixels) and 25 FOVs were acquired in each CLINIcell. Additionally, to determine the ratio of indirect:direct microbubbles in each stock sample, 25 FOVs were acquired using a control CLINIcell without HUVECs every experimental day when a new stock sample of targeted microbubbles was prepared. This control CLINIcell was first blocked with 2% (*w*/*v*) bovine serum albumin (BSA, Sigma-Aldrich, Zwijndrecht, The Netherlands) for 1 h, to avoid nonspecific microbubble binding to the CLINIcell membranes, then washed three times with PBS and preheated to 37 °C before adding the microbubbles (1 × 10^6^ microbubbles/mL final concentration) and placing the CLINIcell in the confocal microscopy imaging set-up.

### 2.6. Binding Efficacy In Vitro under Flow

For experiments under flow, the flow on the HUVECs cultured in the µ-slide was stopped for approximately 10 min to transport the µ-slide from the humidified incubator to the experimental set-up. The µ-slide was first inserted into the set-up upright in a water bath at 37 °C, with the HUVEC monolayer on the bottom of the µ-slide. Then, flow was started at a shear stress of 7.5 dyn/cm^2^ and a mixture of 1.36 µL Hoechst 33342 (10 mg/mL) and 10.9 µL CellMask™ Green Plasma Membrane Stain (5 mg/mL) in 200 µL EGM-2 was injected into the upstream Luer injection port with a 1 mL Luer Solo syringe (Omnifix-F, B Braun, Melsungen, Germany) and 19G needle (Sterican, B Braun). The fluorescent dyes were allowed to incubate under flow for 15 min, after which the µ-slide was inverted to have the HUVEC monolayer on the top side of the µ-slide [20]. For the binding efficacy under flow experiments, different flow conditions were evaluated with a shear stress of 1.25, 2.22, 4.45, 5.0, 6.8, or 7.5 dyn/cm^2^. To monitor binding under flow, confocal microscopy time lapse imaging (0.31 fps) was started 15 s prior to injection of the microbubbles (indirect:direct 1:1 ratio, 1 × 10^6^ microbubbles/mL final concentration) into the downstream Luer injection port and continued for 7 min. Next, to quantify the number of bound microbubbles under flow, single-frame FOVs were acquired at different locations throughout the µ-slide, with each FOV covering 210 × 210 µm (512 × 512 pixels). For 1.25 and 2.22 dyn/cm^2^, the flow was kept constant during imaging of 25 FOVs. For 6.8 and 7.5 dyn/cm^2^, after acquiring 15 FOVs the flow was adjusted to a shear stress of 4.45 or 5 dyn/cm^2^, respectively, and 15 more FOVs were acquired. Additionally, 25 FOVs were acquired in a control µ-slide without HUVECs to determine the ratio of indirect:direct microbubbles on each experimental day. The control µ-slide was connected to the Ibidi pump system and filled with preheated EGM-2 to compare to the situation of µ-slides with HUVECs. The microbubbles (1 × 10^6^ microbubbles/mL final concentration) were injected downstream at a shear stress of 1.5 dyn/cm^2^ to mimic the experimental procedure of the binding efficacy under flow experiment. However, 10 s after injection the flow was turned off to make imaging feasible, since otherwise the unbound microbubbles would float out of the µ-slide in the absence of HUVECs.

### 2.7. Dissociation In Vitro under Flow

For the dissociation under flow experiments, the µ-slide with HUVECs grown under flow was transported to the confocal set-up and dyes were added as described above in Section 2.6. After 15 min incubation of the dyes and subsequent inverting of the µ-slide, the flow was adjusted from 7.5 dyn/cm^2^ to 1.5 dyn/cm^2^. The microbubbles (indirect/direct 1:1 ratio, 1 × 10^6^ microbubbles/mL final concentration) were injected downstream and after 10 s the flow was stopped, to allow the microbubbles to flow into the µ-slide and bind to the HUVEC monolayer. After 5 min incubation with microbubbles, a confocal microscopy time-lapse recording (0.31 fps) was started first without flow, then the flow was started at 1.0 dyn/cm^2^ and gradually increased with 0.5 dyn/cm^2^ steps every 60 s up to 7.5 dyn/cm^2^. The time-lapse FOV covered 210 × 210 µm (512 × 512 pixels) and one time lapse was recorded per µ-slide.

### 2.8. Binding Efficacy In Vivo

For in vivo experiments, the CAM was prepared and dyes were added as described above in Section 2.3. The CLINIcell with the CAM was placed in the confocal set-up in a water bath at 37 °C. Each FOV covered 210 × 210 µm (512 × 512 pixels) and to get a full picture of the blood vessels, *z*-stacks (step-size 0.575 µm) were acquired to include all microbubbles bound at one FOV at different *z*-planes. On average, 12 FOVs were imaged per CAM. Additionally, 25 FOVs were acquired of the stock sample in a control CLINIcell without HUVECs as described above, to determine the indirect:direct ratio of the sample on each experimental day.

### 2.9. Internalization of Bound Microbubbles In Vitro

To image the microbubble-cell morphology in 3D of microbubbles internalized by HUVECs, as described previously (REF: Beekers et al. “The 3D microbubble-cell dynamics: microbubble internalization and drug delivery by pores and tunnels”, under review), CLINIcells were prepared and placed in the confocal imaging set-up as described above. *Z*-stacks were acquired with 0.325 µm steps.

### 2.10. Data Analysis

All in vitro binding efficacy and dissociation under flow results were analyzed with ImageJ software (ImageJ, U.S. National Institutes of Health, Bethesda, MD, USA) by counting the number of direct and indirect microbubbles in each FOV. The in vivo binding efficacy results were analyzed with the NIS-Elements AR Analysis software (version 5.02.00, Nikon Instruments, Amsterdam, The Netherlands) by counting the cumulative number of direct and indirect microbubbles per FOV over all *z*-slices. For the binding efficacy experiments, the median ratio of indirect:direct microbubbles in the control FOVs was calculated per experimental day to correct for any deviations in the stock sample from the desired ratio of 1:1 (indirect:direct). All FOVs with less than five bound direct or indirect microbubbles were excluded from the binding efficacy analysis. The binding efficacy was quantified as the ratio of the number of bound indirect microbubbles divided by the number of bound direct microbubbles (indirect:direct) and corrected for the indirect:direct ratio of the stock sample. The cumulative number of bound microbubbles per µ-slide under flow at 4.45–7.5 dyn/cm^2^ was multiplied by 1.67 to correct for the difference in number of FOVs, since for 1.25–2.22 dyn/cm^2^ 25 FOVs were recorded and for 4.45–7.5 dyn/cm^2^ 15 FOVs were recorded. The results from 4.25 and 5 dyn/cm^2^ were pooled and are presented as 4.7 dyn/cm^2^, and the results from 6.8 and 7.5 dyn/cm^2^ were pooled and are presented as 7.2 dyn/cm^2^.

For the dissociation under flow experiments, for each shear stress the number of bound microbubbles was counted and normalized to the initial number of bound microbubbles, thereby starting at 100% before flow. If the focus was lost for the full 60 s of a shear stress condition during time-lapse imaging, the data point at that shear stress was excluded from analysis. A curve was fitted through the data using the restricted cubic spline curve function (5 knots) in GraphPad Prism 9.0.0. (GraphPad Software, San Diego, CA, USA) Microbubble sizes were measured using the measure Radius tool in the NIS-Elements AR Analysis software (version 5.02.00, Nikon Instruments, Amsterdam, The Netherlands). Microbubbles were only measured if the gas core was clearly visible inside the circular fluorescent coating. For the in vitro binding efficacy, a random sample of 11 FOVs at 1.25 dyn/cm^2^ and 11 FOVs at 2.22 dyn/cm^2^ was analyzed to obtain a minimum N number of 100 microbubbles per type at each flow condition. For 4.45, 5, 6.8, and 7.5 dyn/cm^2^ all FOVs were analyzed. The results from 4.25 and 5 dyn/cm^2^ were pooled and are presented as 4.7 dyn/cm^2^, and the results from 6.8 and 7.5 dyn/cm^2^ were pooled and are presented as 7.2 dyn/cm^2^. For the in vitro dissociation under flow, microbubbles were measured in the first FOV of a time lapse, before starting flow, and at the last FOV, at the maximum flow of 7.5 dyn/cm^2^.

The internalization of bound microbubbles was quantified as described previously (REF Beekers et al. “The 3D microbubble-cell dynamics: microbubble internalization and drug delivery by pores and tunnels”, under review), with a custom semi-automated procedure in MATLAB (The Mathworks Inc., Natick, MA, USA). Briefly, the orthogonal planes crossing the center of the microbubble were found based on the *xy*-plane with maximum fluorescence intensity in the DiI (direct microbubble) or DiD (indirect microbubble) channel. These orthogonal planes, i.e., *xz*- and *yz*-planes, were then used to obtain the microbubble location by fitting a circle through the maximum intensity in the DiI or DiD channel. The CellMask Green channel of the same orthogonal planes was then used to identify the apical and basal cell membrane at the location of the microbubble. These planes were used to quantify the cell thickness. Next, the internalized depth was quantified at the center of the microbubble, as the difference between the *z*-plane of the apical cell membrane and the bottom of the microbubble (i.e., *z*-plane closest to the basal cell membrane). Finally, the CellMask Green channel was scored manually for the occurrence of a dome, that is when the top of the microbubble was covered by the cell membrane, and the occurrence of green rings, that is when there was a ring with high fluorescence intensity present at the location of the microbubble.

### 2.11. Statistics

The statistical analysis was done using IBM SPSS Statistics 25 (IBM, Armonk, New York, NY, USA). First a Shapiro–Wilk test was used to assess the normality and distribution of the data. As the data was non-parametric, a Mann–Whitney U test or Kruskal–Wallis test was used to test for differences between groups and the exact Wilcoxon Signed-Rank test was used for paired data (microbubble size from Coulter Counter measurement and percentage of bound microbubbles in dissociation under flow experiment). The correlation between parameters was assessed with Pearson’s correlation test and differences between groups were only tested for *N* > 2.

## 3. Results

**Figure 1:**
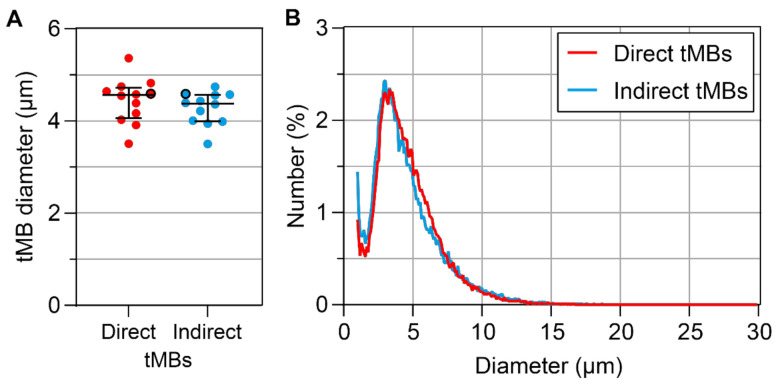
(**A**) Number weighted mean diameter (µm) of direct and indirect targeted microbubbles (tMBs). Each circle represents one sample of targeted microbubbles (*N* = 12) for each experimental day, with the median and interquartile range (IQR) overlaid in black. The size distribution of circles with a black border is shown in (**B**): number weighted size distribution of direct (red) and indirect (blue) targeted microbubbles.

In Figure 1, the mean microbubble diameters and size distribution of direct and indirect targeted microbubbles are summarized. There was no significant difference between the mean size of direct and indirect targeted microbubbles (*p* = 0.209, Figure 1A). The size distribution was polydisperse and comparable for both types of microbubbles, as shown in Figure 1B.

**Figure 2:**
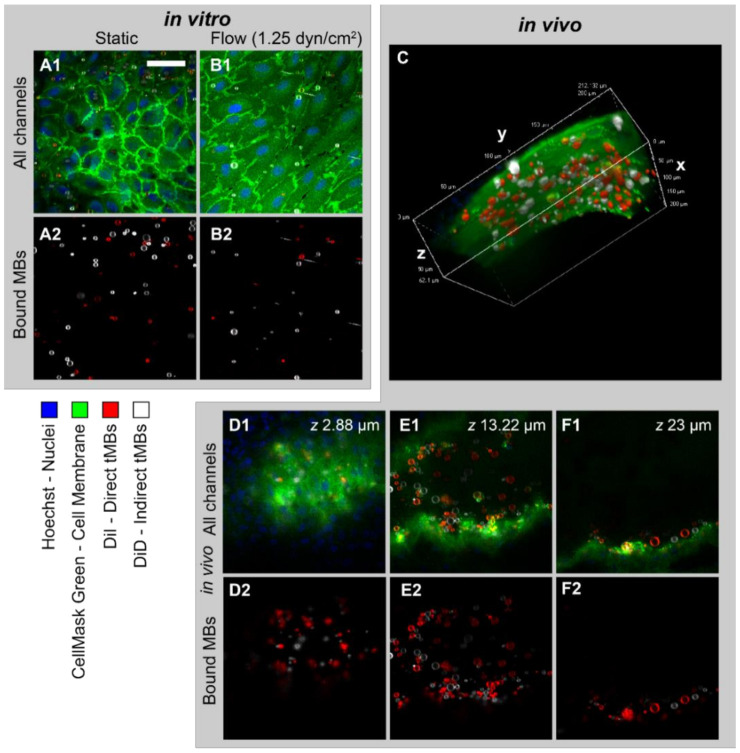
Typical examples of targeted microbubbles bound to a monolayer of HUVECs cultured (**A1**,**A2**) statically or (**B1**,**B2**) under flow (cultured at 7.5 dyn/cm^2^, imaged at 1.25 dyn/cm^2^) showing (**A1**,**B1**) all channels and (**A2**,**B2**) DiI and DiD channels. (**C**) Volume view of targeted microbubbles bound to endothelial cells in vivo in the CAM model based on confocal *z*-stack. (**D1**–**F1**,**D2**–**F2**) 2D view of slices at different *z* positions in *z*-stack shown in (**C**) and imaged from the top, showing (**D1**–**F1**) all channels and (**D2**–**F2**) DiI and DiD channels. Cell membrane shown in green, cell nuclei in blue, direct microbubbles in red (DiI) and indirect microbubbles in white (DiD). Scale bar represents 50 µm and applies to all 2D images. Normalized ratio of indirect/direct bound microbubbles was (**A1**,**A2**) 1.87, (**B1**,**B2**) 3.09, (**C**) 1.14. All experiments were performed in a temperature-controlled water bath at 37 °C.

The binding efficacy of direct and indirect targeted microbubbles was evaluated in three models using confocal imaging of bound microbubbles, as shown in Figure 2. HUVECs grown under static conditions (Figure 2(A1,A2)) had a typical cobblestone appearance, with high fluorescence intensity at the cell–cell borders. In contrast, the HUVECs grown under flow (shear stress 7.5 dyn/cm^2^, Figure 2(B1,B2)) were more elongated and covered a larger area per cell. The DiI and DiD channels showing the bound direct and indirect microbubbles in red and white, respectively, are presented in Figure 2(A2,B2). The white stripes in Figure 2(B2) represent nonbound microbubbles flowing through the µ-channel during imaging. Figure 2C shows the volume view of a *z*-stack acquired of a vessel in the in vivo CAM model. The curved top side of the vessel is visible with targeted microbubbles (red and white) bound to the endothelial cells in green. Three slices from the *z*-stack are shown in Figure 2(D1–F1), with the bound microbubbles in Figure 2(D2–F2). In comparison to the HUVEC monolayers, the cell–cell borders were less distinguishable in the CAM model due to the more challenging imaging environment and 3D structure in vivo. The mean heart rate was 88 ± 24 beats per minute.

**Figure 3:**
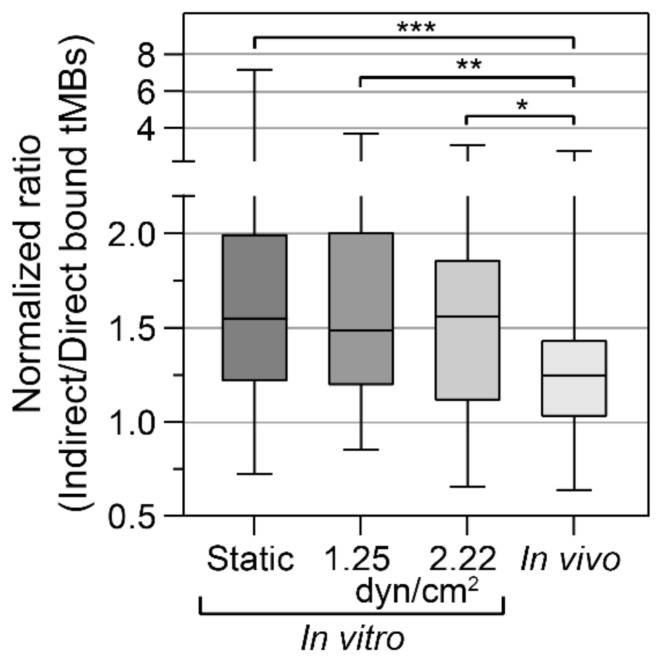
Binding efficacy of direct versus indirect targeted microbubbles (tMBs), plotted as the ratio of indirect:direct bound microbubbles per condition, normalized to the median ratio (indirect:direct microbubbles) of the control without cells. Static (*N* = 186 FOVs), shear stress 1.25 dyn/cm^2^ (*N* = 47 FOVs), shear stress 2.22 dyn/cm^2^ (*N* = 23 FOVs), and in vivo in the CAM model (*N* = 66 FOVs). Boxplots show the median, IQR and whiskers from minimum to maximum. Statistical significance is indicated with * *p* < 0.05, ** *p* < 0.01, *** *p* < 0.001. Only results from FOVs with >5 bound direct and >5 bound indirect microbubbles are included.

The binding efficacy was quantified as the normalized ratio of indirect to direct bound microbubbles in vitro under static conditions and under flow (1.25 and 2.22 dyn/cm^2^), and in vivo in the CAM model, as presented in Figure 3. Since all ratios based on FOVs with less than 5 bound microbubbles were excluded, results from higher shear stresses in vitro (4.7 to 7.2 dyn/cm^2^) are not presented in Figure 3. The mean ratio in the stock sample was (0.85 ± 0.25):1 indirect:direct targeted microbubbles (*N* = 12 experimental days). For the static condition, more indirect than direct microbubbles were bound in 89.2% of the FOVs, with a median of 1.55× more indirect microbubbles bound. There was some variability in the extent of how many more indirect microbubbles were bound, ranging from 0.72 to 7.2× the number of direct-bound microbubbles. The cumulative number of microbubbles bound in 25 FOVs, i.e., one CLINIcell, ranged from 1000 to 2000. For the flow condition with 1.25 dyn/cm^2^, 87.2% of the FOVs had more indirect than direct microbubbles bound, with a median of 1.49× more indirect microbubbles bound, and for the flow condition with 2.22 dyn/cm^2^, 82.6% of the FOVs had more indirect than direct microbubbles bound with a median of 1.56× more indirect microbubbles bound. Both in vitro flow shear stresses resulted in ratios comparable to the static condition but with a lower maximum and lower standard deviation. Finally, using the in vivo CAM model, in 77.3% of the FOVs more indirect than direct microbubbles had bound with a median of 1.25× more indirect microbubbles bound. In vivo, most of the microbubbles bound in the vein near the injection site and no bound microbubbles were observed in the arteries. The cumulative number of microbubbles bound per chicken embryo ranged from 150 to 1800. The ratio of indirect:direct bound microbubbles in static conditions was significantly higher than in vivo (*p* < 0.001). Furthermore, the ratios at 1.25 and 2.22 dyn/cm^2^ for the in vitro flow conditions were significantly higher than in vivo (*p* = 0.001 and *p* = 0.031, respectively).

**Figure 4:**
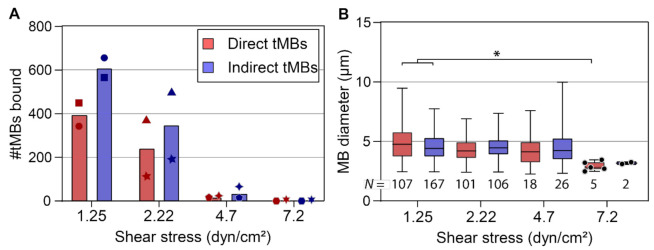
Direct and indirect targeted microbubbles (tMBs) bound in vitro at different shear stresses. (**A**) Mean cumulative number of microbubbles counted in a total of 25 FOVs per slide, each symbol represents one slide. Bar represents mean of *N* = 2 µ-slides. (**B**) Microbubble diameter as a function of shear stress. *N* = number of microbubbles measured, in 11 FOVs for 1.25 and 2.22 dyn/cm^2^ and all FOVs for 4.7 and 7.2 dyn/cm^2^. Boxplots are presented with median, IQR, and whiskers from min to max. For *N* < 6 individual data points are shown as circles. Statistical significance is indicated with * *p* < 0.05. Results from 4.25 and 5 dyn/cm^2^ were pooled presented as 4.7 dyn/cm^2^, results from 6.8 and 7.5 dyn/cm^2^ were pooled presented as 7.2 dyn/cm^2^.

Binding efficacy under flow was investigated in vitro at shear stresses of 1.25 and 2.22 as shown in Figure 3, and also at 4.7 and 7.2 dyn/cm^2^. With increasing shear stress, the number of microbubbles binding under flow decreased towards 0 at 7.2 dyn/cm^2^. The cumulative number of bound microbubbles per µ-slide is shown in Figure 4A, to incorporate the results from high-shear stress conditions (>2.22 dyn/cm^2^) in which only a limited number of microbubbles bound under flow. Despite the variability in numbers of bound microbubbles between µ-slides at the same shear stress, the number of bound indirect microbubbles was higher than the number of bound direct microbubbles at all shear stresses except for 7.2 dyn/cm^2^. The diameters of bound microbubbles were measured to investigate the influence of shear stress on the size distribution of microbubbles binding under flow (Figure 4B). *N* numbers in Figure 4B do not correspond to the cumulative numbers from Figure 4A, as not every microbubble had a clearly visible gas core and thus some microbubbles were counted without measuring the diameter. The mean diameter of bound microbubbles was comparable at 1.25 to 4.7 dyn/cm^2^, while the diameter of bound direct microbubbles at 7.2 dyn/cm^2^ was significantly smaller than the diameter of bound direct (*p* = 0.011) and indirect (*p* = 0.023) microbubbles at 1.25 dyn/cm^2^. The sample size of bound indirect microbubbles at 7.2 dyn/cm^2^ (*N* = 2) was too small for statistical comparison to the other groups.

**Figure 5:**
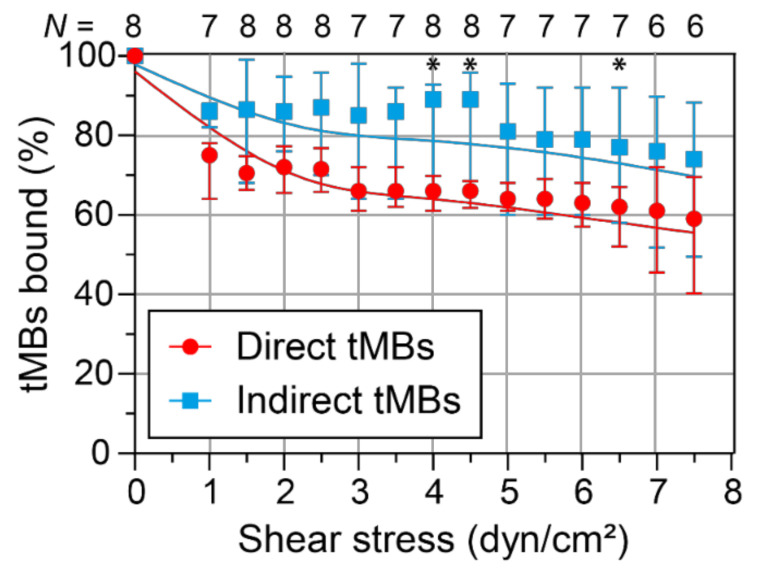
Dissociation of bound targeted microbubbles (tMBs) at increasing shear stress in vitro. The median percentage of bound direct (red) and indirect (blue) microbubbles, relative to the number of microbubbles bound before flow, is plotted with the IQR and curve fitting (restricted cubic spline). Statistical significance is indicated with * *p* < 0.05. The number of µ-slides analyzed at each shear stress is shown on top of the graph as *N* number.

The binding dissociation under flow was investigated in vitro by allowing targeted microbubbles to bind to a HUVEC monolayer without flow and subsequent exposure to increasing shear stress conditions. The median number of direct microbubbles bound before flow in one FOV was 63 (interquartile range (IQR) 32–139) and the median number of indirect microbubbles bound was 49 (IQR 25–91). After correction for the stock sample, this corresponded to a ratio of 1.13 ± 0.32 indirect/direct targeted microbubbles (mean ± SD, *N* = 8). Figure 5 shows the percentage of targeted microbubbles, relative to the number of microbubbles bound before flow, remaining bound to the HUVEC monolayer upon increasing shear stress with a spline curve fitted through the data. The median percentage of indirect microbubbles bound was always higher than the median percentage of direct microbubbles, with a significant difference at 4, 4.5, and 6.5 dyn/cm^2^. Based on the curve fitting, there was a significant difference in dissociation rate between direct and indirect microbubbles at low shear stresses (1 to 3 dyn/cm^2^), with the slope being −7.5 (95% confidence interval: −8.0–−7.0) for direct microbubbles and −4.7 (95% confidence interval: −5.0–−4.4) for indirect microbubbles. No differences in microbubble size distribution were found after evaluation of the microbubble sizes before flow and at 7.5 dyn/cm^2^ (Supplementary Figure S1).

**Figure 6:**
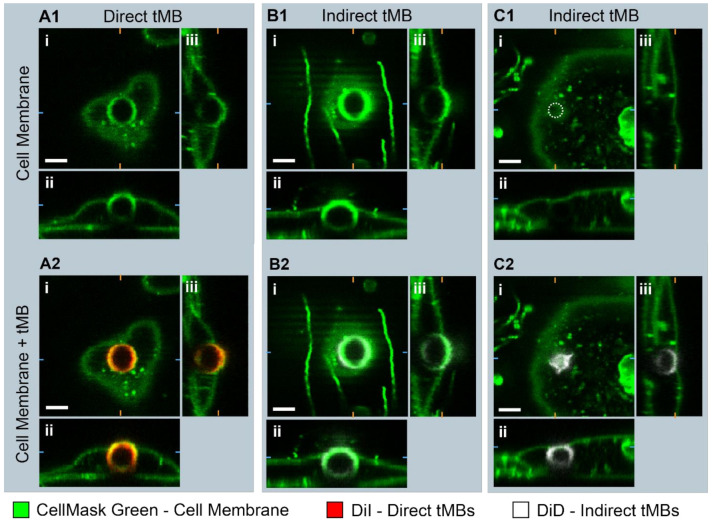
Orthogonal views of confocal microscopy *z*-stack of (**A1**,**A2**) bound direct microbubble (diameter 6.0 µm, internalized depth 5.5 µm), (**B1**,**B2**) bound indirect microbubble (diameter 5.8 µm, internalized depth 4.7 µm), and (**C1**,**C2**) bound indirect microbubble (diameter 3.7 µm, internalized depth 4.4 µm) with microbubble location marked with white dotted circle in C1. Side views were imaged with the objective from (**ii**) above or (**iii**) the right. Scale bar represents 5 µm, orange and blue markings indicate the cross-section of the orthogonal planes of the side views.

To evaluate the internalization of direct and indirect bound microbubbles, the 3D morphology of targeted microbubbles bound to HUVECs was studied in static conditions with confocal *z*-stacks as shown in Figure 6. In Figure 6(A1,B1) a bright fluorescent ring can be observed in the middle of the image in the CellMask green channel, at the location of the internalized direct and indirect microbubble, respectively. Furthermore, the apical cell membrane in green is covering the top of the internalized microbubbles, referred to as a dome. In Figure 6(C1,C2), an example is shown of an internalized indirect microbubble where a dome is present but no green fluorescent ring.

**Figure 7:**
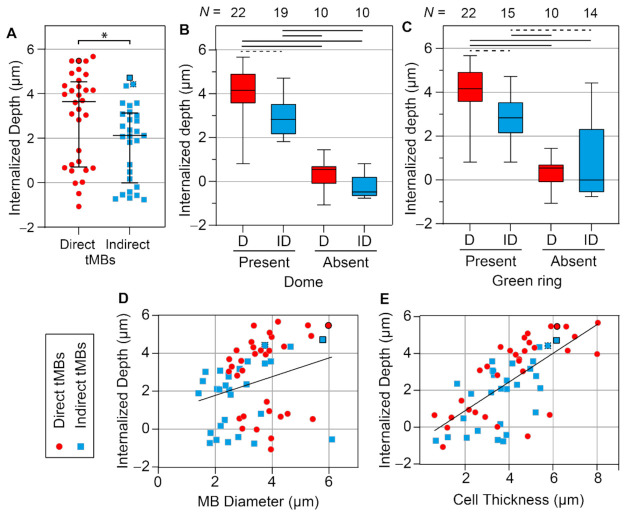
Internalization of targeted microbubbles (tMBs) bound to a HUVEC monolayer in static conditions with direct (circles) in red and indirect (squares) in blue. (**A**) Internalized depth (µm) of direct (*N* = 32) versus indirect (*N* = 29) targeted microbubbles with median and IQR. Significance is indicated with * *p* < 0.05. (**B**,**C**) Internalized depth of direct (**D**, red) and indirect (ID, blue) bound microbubbles with (**B**) dome or (**C**) green ring present or absent in the CellMask Green channel, shown as boxplot with whiskers from min to max and N numbers listed above the graphs. Statistics are indicated with a solid black line for *p* < 0.001 and a dashed line for *p* < 0.01. (**D**) Internalized depth as a function of microbubble diameter. (**E**) Internalized depth as a function of cell thickness. In (**A**,**D**,**F**) the examples from Figure 6 are marked with: F6A) black circle, F6B) black square, and F6C) dotted black square.

Quantification of the internalized depth is presented in Figure 7. The internalized depth of direct targeted microbubbles was significantly larger than that of indirect targeted microbubbles (*p* = 0.027, Figure 7A). Moreover, the size of direct microbubbles (median diameter 3.7 µm) was significantly larger (*p* < 0.001) than the size of indirect microbubbles (median diameter 2.8 µm) analyzed for internalized depth. All microbubbles analyzed for internalized depth (*N* = 61 in total) were also scored for the presence of a dome and a green ring in the cell membrane. The internalized depth of microbubbles with a dome (*N* = 41) was significantly (*p* < 0.001, Supplementary Figure S2A) larger than that of microbubbles without a dome (*N* = 20). Furthermore, the internalized depth of microbubbles with a green ring (*N* = 37) was significantly (*p* < 0.001, Supplementary Figure S2B) larger than that of microbubbles without a green ring (*N* = 24). Not all microbubbles covered by the apical cell membrane, classified as a dome, also displayed a green ring (*N* = 4, Figure 6C). However, in all cases where a green ring was present in the CellMask Green channel, a dome was also found (*N* = 37). The influence of ligand distribution on the occurrence of domes and green rings was further investigated by comparing the direct and indirect targeted microbubbles (Figure 7B,C). For microbubbles with a dome, the internalized depth of direct microbubbles (*N* = 22) was significantly higher (*p* = 0.001) than that of indirect microbubbles (*N* = 19). For microbubbles with a green ring, the internalized depth of direct microbubbles (*N* = 22) was significantly higher (*p* = 0.001) than that of indirect microbubbles (*N* = 15). There were no significant differences in internalized depth of direct (*N* = 10) and indirect (*N* = 10) microbubbles without a dome (*p* = 0.059) and without a green ring (direct *N* = 10, indirect *N* = 14, *p* = 0.861). The correlation of internalized depth with microbubble size and cell thickness was first analyzed per microbubble type. Based on linear regression, no significant differences were found between the slopes and intercepts of the lines for direct and indirect microbubbles; therefore, the data were pooled for correlation analysis. The internalized depth was positively correlated with the microbubble size (Spearman correlation coefficient *ρ* = 0.278, *p* = 0.030, Figure 7D). Additionally, the internalized depth was positively correlated with the cell thickness (*ρ* = 0.686, *p* < 0.001, Figure 7E).

**Figure 8:**
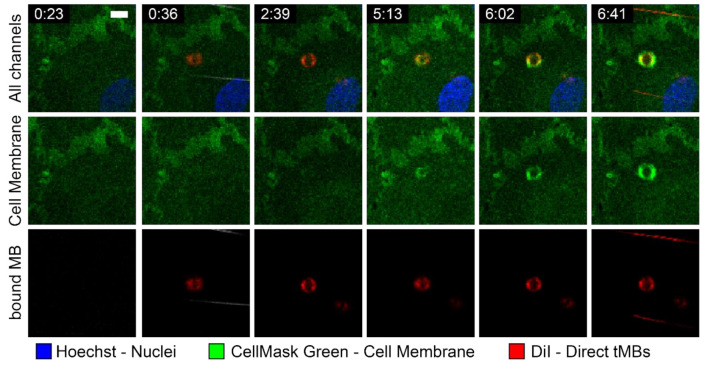
Internalization event of targeted direct microbubble (diameter 5.9 µm) over time indicated with min:sec, at a shear stress of 1.25 dyn/cm^2^ and with targeted microbubbles added at 0:15. Scale bar represents 10 µm and applies to all images.

With time-lapse imaging of targeted microbubbles binding under flow to a HUVEC monolayer, an internalization event could be tracked over time, as shown in Figure 8. At 0:36 a direct targeted microbubble appeared in the field of view and bound to the HUVEC monolayer. Over the next 6 minutes a bright green ring appeared in the cell membrane at the location of the bound microbubble, indicating that the bound microbubble was being internalized by the HUVEC.

## 4. Discussion

The effect of ligand distribution on the binding efficacy of α_ν_β_3_-targeted microbubbles was evaluated by comparing direct and indirect targeted DSPC-based microbubbles with heterogeneous and homogeneous ligand distributions, respectively. In the three experimental models used for this study—a HUVEC monolayer under static conditions and under flow with a shear stress of 1.25 to 7.5 dyn/cm^2^, and the in vivo CAM model—microbubbles with a homogeneous ligand distribution always had a higher binding efficacy than those with a heterogeneous ligand distribution. Furthermore, the dissociation rate of microbubbles with a homogeneous ligand distribution was significantly lower and more microbubbles with a homogeneous ligand distribution remained bound at a shear stress of 1.5 to 7.5 dyn/cm^2^. Additionally, the effect of ligand distribution on internalization of α_ν_β_3_-targeted microbubbles was evaluated by quantifying the internalized depth of bound direct and indirect microbubbles to a HUVEC monolayer in static conditions, and was found to be independent of the ligand distribution.

The ratio of indirect to direct bound microbubbles was calculated as a measure of binding efficacy and found to be >1, indicating that more indirect targeted microbubbles had bound. Therefore, the indirect microbubbles, with a homogeneous ligand distribution, had a higher binding efficacy than the direct microbubbles, with a heterogeneous ligand distribution. There were no significant differences in microbubble size; therefore any differences in binding efficacy between direct and indirect targeted microbubbles cannot be caused by a difference in size. Although our previous study found a slight difference in size between direct and indirect microbubbles [34], the microbubbles in that study were washed twice whereas the targeted direct and indirect microbubbles in the present study underwent five washing steps before measuring the size distribution. Washing of microbubbles by centrifugation is known to influence the size distribution [44], which may explain why the more size-selected direct and indirect targeted microbubbles in the present study had no significant difference in size. The variability in binding efficacy was the highest without flow, when microbubbles bound to a HUVEC monolayer under static conditions. There was no correlation between the cumulative number of microbubbles bound per CLINIcell, ranging from 1000 to 2000 in 25 FOVs, and the percentage of indirect bound microbubbles, suggesting that the variability in binding efficacy was not due to a difference in total number of bound microbubbles per CLINIcell. In addition, there was no correlation between the percentage of indirect bound microbubbles and the passage number of the HUVECs used. Regardless of the variability in the binding efficacy under static conditions, the normalized ratio of indirect:direct bound microbubbles was > 1 in 89.2% of the FOVs, indicating that microbubbles with a homogeneous ligand distribution had a higher binding efficacy than microbubbles with a heterogeneous ligand distribution in static conditions.

The next step was to evaluate the binding efficacy and dissociation rate of α_ν_β_3_-targeted microbubbles under flow, in order to mimic more physiologically relevant conditions. Shear stresses of 1 to 7.5 dyn/cm^2^ were investigated, comparable to the blood flow in healthy veins [45]. The target α_ν_β_3_ integrin is expressed during angiogenesis [46] and atherosclerosis [47], and is an established target for molecular imaging of tumors [48] and antitumor therapies [49]. Although tumors are known to have a complex microenvironment with highly variable blood flow, shear stresses have been estimated to be as low as 0.5 dyn/cm^2^ [50,51]. The HUVEC monolayers cultured under flow (shear stress 7.5 dyn/cm^2^) in this study had a different morphology compared to the HUVEC monolayers cultured under static conditions, indicating that the cells had adapted in response to the shear stress to which they were exposed, in agreement with previous observations [52]. Although this was not investigated in the present work, previous studies reported that the α_ν_β_3_ integrin expression was upregulated when HUVECs [53] and bovine endothelial cells [54] were cultured under flow, in contrast to cells cultured under static conditions. Although all experiments were performed at 37 °C and thus can be extrapolated to the in vivo situation, different ambient hydrostatic pressures and temperatures were not investigated in relation to microbubble binding characteristics. What is known is that microbubbles’ acoustic properties change dependent on the ambient hydrostatic pressure, where the subharmonic amplitude decreased with increasing pressure [55], and temperature, where the shell elasticity and viscosity decreased [56,57] and attenuation either decreased [58] or increased [57,59] with increasing temperature. In addition, temperature changes may affect microbubble sizes, as decreases [58], increases [59,60], and no effect [57,58] in size have been reported. An increase in size will affect microbubble binding efficacy at higher shear rates as shown in our study (Figure 4B).

When looking at the cumulative number of bound microbubbles in all 25 FOVs per µ-slide (Figure 4), always more indirect microbubbles bound than direct microbubbles. This indicates that α_ν_β_3_-targeted microbubbles with a homogeneous ligand distribution bound more efficiently under flow than those with a heterogeneous ligand distribution, which was confirmed by the ratio of indirect:direct bound microbubbles (Figure 3). At 1.25 dyn/cm^2^, the cumulative number of bound microbubbles was comparable to the number of bound microbubbles under static conditions, being approximately 1000 bound microbubbles per 25 FOVs (Figure 4A). The number of microbubbles binding under flow decreased with increasing shear stress, which is in agreement with previous studies on α_ν_β_3_-targeted microbubbles binding to HUVECs under flow (shear stress 0.5–5 dyn/cm^2^) [61], and microbubbles targeted to other biomarkers such as P-selectin [62], vascular cell adhesion molecule (VCAM)-1 [63], and intracellular adhesion molecule (ICAM)-1 [64], all using microbubbles functionalized with an antibody through biotin–streptavidin coupling. In our study, the size distribution of bound microbubbles at 1.25 to 4.7 dyn/cm^2^ was comparable, suggesting that at a low shear stress the microbubble size has limited influence on the binding rate. In contrast, at 7.2 dyn/cm^2^ the microbubble diameter of bound direct microbubbles, median 3.11 µm (IQR 2.77–3.23), was significantly smaller than at 1.25 dyn/cm^2^. While it should be noted that the sample sizes of bound microbubbles at 7.2 dyn/cm^2^ were small, this could be an indication that smaller microbubbles have a higher binding rate at a high shear stress. A computational model revealed that a diameter of 2 to 4 µm would be the optimal size for microbubbles targeted to P-selectin and E-selectin [65], and a study on targeted spherical particles (diameter 0.1 to 10 µm) showed that spheres with 2 to 5 µm diameter were optimal for targeting under flow [66]. This may be explained by the increased force encountered by larger particles, as they extend farther from the endothelial cell layer to the center of the vessel.

The dissociation under flow was investigated by allowing the microbubbles to bind first and then starting an escalating flow regime with shear stresses from 1 to 7.5 dyn/cm^2^. The measured dissociation rate of direct microbubbles was higher than that of indirect microbubbles at low shear stresses (1–5 dyn/cm^2^), although above 5 dyn/cm^2^ the dissociation rates of direct and indirect microbubbles were similar. The difference between direct and indirect microbubbles in percentage of bound microbubbles was significant at 4, 4.5, and 6.5 dyn/cm^2^; however, with a larger sample size a significant difference between direct and indirect microbubbles may also be expected for other shear stresses above 4.5 dyn/cm^2^. There were no differences in size distribution of microbubbles bound before flow started and at 7.5 dyn/cm^2^, indicating that although the binding rate may be influenced by the microbubble size, the dissociation of bound microbubbles occurred independent of the size. Moreover, the mean size of bound microbubbles was similar to the mean size measured by Coulter Counter. Together, these results confirm that under flow a homogeneous ligand distribution results in a higher binding efficacy than a heterogeneous ligand distribution.

A study on P-selectin-targeted microbubbles reported that microbubble dissociation or detachment under flow was related to the receptor density on the target surface [62], with our results corresponding to the dissociation rate of the highest receptor density tested (109 molecules/µm^2^). In our study, the percentage of microbubbles remaining bound at increasing shear stress was several times higher than the percentage reported recently on vancomycin-decorated microbubbles binding to a bacterial biofilm under flow [20]. With the experimental set-up used for the present study, the unbound targeted microbubbles kept on circulating through the µ-slide with the HUVEC monolayer for as long as the flow was on. Hence, it is possible that at low shear stresses new microbubbles bound during the experiment and the results do not only reflect a difference in dissociation rate, but also a difference in binding of microbubbles at low shear stresses. The number of microbubbles remaining bound at shear stresses above 4 dyn/cm^2^ was much higher than the number of microbubbles binding under flow at 4.7 and 7.2 dyn/cm^2^, which is in concert with previous reports on P-selectin-targeted microbubbles [62]. This indicates that while the binding rate may be low at high shear stress, the microbubbles can remain bound successfully. To improve the binding rate in high shear stress conditions, acoustic radiation forces can be used to reduce the flow speed of targeted microbubbles and displace them towards the target surface [67].

Finally, the binding efficacy of α_ν_β_3_-targeted microbubbles was evaluated in vivo using the CAM model. Although the size of the injected vein and the number of microbubbles injected varied, the number of indirect bound microbubbles was higher than the number of direct bound microbubbles in 77.3% of the FOVs, with a median of 1.25× more indirect bound microbubbles. Studies have shown that for ultrasound molecular imaging, the acoustic scattering of individual bound microbubbles can be detected [39]. Based on this, using indirect targeted microbubbles—with homogeneous ligand distribution—would result in 25% higher molecular signal in vivo compared to the direct targeted microbubbles, with heterogeneous ligand distribution. The experimental set-up limited the view of the CAM; hence the injection site could not be imaged in each chicken embryo. Nonetheless, this should have no effect on the ratio of indirect:direct bound microbubbles. A study using intravital microscopy to observe the behavior of targeted microbubbles in the microcirculation of rodents reported that microbubbles predominantly bound in veins and not the arteries or arterioles [68], similar to our observation in the present study. The observed heart rates of the chicken embryos were in agreement with a previous report on chicken embryo experiments involving α_ν_β_3_-targeted microbubbles, where it was found that the uptake of a model drug was increased when more microbubbles bound to the endothelial cells [40]. In the context of our results, this suggests that the use of targeted microbubbles with homogeneous ligand distribution may result in increased enhancement of drug delivery compared to those with a heterogeneous ligand distribution.

The effect of ligand distribution on the internalization of α_ν_β_3_-targeted microbubbles was investigated, since a recent study has shown how the drug delivery outcome upon sonoporation is affected by the internalization of targeted microbubbles (REF Beekers et al. “The 3D microbubble-cell dynamics: microbubble internalization and drug delivery by pores and tunnels”, under review). While there was a significant difference in internalized depth between direct and indirect microbubbles, the internalized depth was size-dependent and a significant difference was found in the diameters of direct and indirect microbubbles in this data set. Therefore, the difference found in internalized depth is likely due to a difference in microbubble size, as opposed to the difference in ligand distribution. The internalized depth and its correlations with microbubble diameter and cell thickness reported here are all in agreement with the previously published results on indirect microbubbles, although the number of non-internalized indirect microbubbles found here was slightly higher than in the previous study. The dome formation and occurrence of green rings is substantiated by the previous study as well. For both types of microbubbles, two separate groups were observed: those with internalized depth between 2 to 6 µm and those with internalized depth around 0 µm (Figure 6A). This separation into two groups was confirmed by the dome formation, based on which the microbubbles could be divided into a non-internalized group without dome and an internalized group of microbubbles with a dome. In all cases where a green ring was observed in the cell membrane, the microbubble was internalized, and hence a green ring was a clear marker for internalization. On the other hand, a green ring was not always present when a microbubble was internalized. It is possible that the occurrence of bright fluorescent rings at the location of the bound microbubbles is caused by rearrangement of the cell membrane lipids and fluorescent dye. The process of internalization appeared to be independent of the ligand distribution, and is instead expected to be receptor-mediated (REF Beekers et al. “The 3D microbubble-cell dynamics: microbubble internalization and drug delivery by pores and tunnels”, under review).

A live internalization event was observed with a direct targeted microbubble under flow, based on the emerging green fluorescent ring observed in the cell membrane (Figure 7). Since the confocal microscopy imaging of microbubbles binding under flow was all in 2D, it was not possible to quantify the internalized depth. The internalization of the microbubble took approximately 6 min, the same time scale found in studies on internalization of 3.2 µm sized silicon micro-particles, which were starting to be encapsulated by the HUVEC cell membrane after 5 min of incubation and were fully internalized after 15 min [69]. Furthermore, non-targeted phospholipid-coated microbubbles with diameters from 2.8 to 4.1 µm adhered to activated leukocytes after 3 min incubation and were fully phagocytosed, i.e., internalized, after 15 min of incubation [70]. These microbubbles were internalized through complement opsonization; however, in our previous study non-targeted microbubbles were not internalized by HUVECs (REF Beekers et al. “The 3D microbubble-cell dynamics: microbubble internalization and drug delivery by pores and tunnels”, under review). Therefore, it is unlikely that complement factors binding to the microbubble surface or ligand play a role in the internalization of microbubbles by HUVECs as studied here.

## 5. Conclusions

The effect of the ligand distribution on the binding efficacy of α_ν_β_3_-targeted microbubbles was evaluated in vitro under static conditions and under flow with a shear stress from 1.25 to 7.5 dyn/cm^2^, and in vivo in the CAM model. Microbubbles with a homogeneous ligand distribution had a higher binding efficacy than those with a heterogeneous ligand distribution. The dissociation rate of bound microbubbles with homogeneous ligand distribution was significantly lower than the dissociation rate of those with heterogeneous ligand distribution at a low shear stress (1 to 5 dyn/cm^2^). Although the ligand distribution had no influence on the internalization of α_ν_β_3_-targeted microbubbles by HUVECs, the internalization depth correlated with the microbubble size and cell thickness. In conclusion, the ligand distribution of targeted microbubbles has a significant effect on the binding efficacy in vitro and in vivo. For optimal results in ultrasound molecular imaging and drug delivery with targeted microbubbles, the indirect production method of phospholipid-coated microbubbles is preferred, whereby the phospholipid components are first dissolved in organic solvent to obtain a lipid film before the lipids are dispersed in aqueous medium to achieve a homogeneous ligand distribution.

## Data Availability

The data presented in this study are available on request from the corresponding author. The data are not publicly available due to the complex nature and tailored analysis for the experimental set-ups used in this study.

## Supplementary Materials

The following supporting information can be downloaded at: https://www.mdpi.com/article/10.3390/pharmaceutics14020311/s1, Figure S1: Diameter of bound direct and indirect targeted microbubbles from dissociation under flow assay; Figure S2: Internalized depth of bound targeted microbubbles with a dome or green ring.

## References

[1] Klibanov A.L., Krause W. (2002). Ultrasound Contrast Agents: Development of the Field and Current Status. Contrast Agents II: Optical, Ultrasound, X-ray and Radiopharmaceutical Imaging.

[2] Chong W.K., Papadopoulou V., Dayton P.A. (2018). Imaging with ultrasound contrast agents: Current status and future. Abdom. Radiol..

[3] Azmin M., Harfield C., Ahmad Z., Edirisinghe M., Stride E. (2012). How do microbubbles and ultrasound interact? Basic physical, dynamic and engineering principles. Curr. Pharm. Des..

[4] van Wamel A., Kooiman K., Harteveld M., Emmer M., ten Cate F.J., Versluis M., de Jong N. (2006). Vibrating microbubbles poking individual cells: Drug transfer into cells via sonoporation. J. Control Release.

[5] Beekers I., Vegter M., Lattwein K.R., Mastik F., Beurskens R., van der Steen A.F.W., de Jong N., Verweij M.D., Kooiman K. (2020). Opening of endothelial cell–cell contacts due to sonoporation. J. Control. Release.

[6] Fekri F., Delos Santos R.C., Karshafian R., Antonescu C.N. (2016). Ultrasound Microbubble Treatment Enhances Clathrin-Mediated Endocytosis and Fluid-Phase Uptake through Distinct Mechanisms. PLoS ONE.

[7] Zhang J., Wang S., Deng Z., Li L., Tan G. (2018). Ultrasound-Triggered Drug Delivery for Breast Tumor Therapy Through iRGD-Targeted Paclitaxel-Loaded Liposome-Microbubble Complexes. J. Biomed. Nanotechnol..

[8] Snipstad S., Berg S., Mørch Ý., Bjørkøy A., Sulheim E., Hansen R., Grimstad I., van Wamel A., Maaland A.F., Torp S.H. (2017). Ultrasound Improves the Delivery and Therapeutic Effect of Nanoparticle-Stabilized Microbubbles in Breast Cancer Xenografts. Ultrasound Med. Biol..

[9] Dimcevski G., Kotopoulis S., Bjånes T., Hoem D., Schjøtt J., Gjertsen B.T., Biermann M., Molven A., Sorbye H., McCormack E. (2016). A human clinical trial using ultrasound and microbubbles to enhance gemcitabine treatment of inoperable pancreatic cancer. J. Control. Release.

[10] Hynynen K., McDannold N., Vykhodtseva N., Jolesz F.A. (2001). Noninvasive MR imaging-guided focal opening of the blood-brain barrier in rabbits. Radiology.

[11] Meng Y., Reilly R.M., Pezo R.C., Trudeau M., Sahgal A., Singnurkar A., Perry J., Myrehaug S., Pople C.B., Davidson B. (2021). MR-guided focused ultrasound enhances delivery of trastuzumab to Her2-positive brain metastases. Sci. Transl. Med..

[12] Engelhardt K., Rumpel A., Walter J., Dombrowski J., Kulozik U., Braunschweig B., Peukert W. (2012). Protein Adsorption at the Electrified Air–Water Interface: Implications on Foam Stability. Langmuir.

[13] Khan A.H., Dalvi S.V. (2020). Kinetics of albumin microbubble dissolution in aqueous media. Soft Matter.

[14] Bloch S.H., Wan M., Dayton P.A., Ferrara K.W. (2004). Optical observation of lipid- and polymer-shelled ultrasound microbubble contrast agents. Appl. Phys. Lett..

[15] Lindner J.R., Song J., Christiansen J., Klibanov A.L., Xu F., Ley K. (2001). Ultrasound Assessment of Inflammation and Renal Tissue Injury With Microbubbles Targeted to P-Selectin. Circulation.

[16] Klibanov A.L., Hughes M.S., Villanueva F.S., Jankowski R.J., Wagner W.R., Wojdyla J.K., Wible J.H., Brandenburger G.H. (1999). Targeting and ultrasound imaging of microbubble-based contrast agents. Magma.

[17] Wischhusen J., Wilson K.E., Delcros J.G., Molina-Peña R., Gibert B., Jiang S., Ngo J., Goldschneider D., Mehlen P., Willmann J.K. (2018). Ultrasound molecular imaging as a non-invasive companion diagnostic for netrin-1 interference therapy in breast cancer. Theranostics.

[18] Abou-Elkacem L., Wang H., Chowdhury S.M., Kimura R.H., Bachawal S.V., Gambhir S.S., Tian L., Willmann J.K. (2018). Thy1-Targeted Microbubbles for Ultrasound Molecular Imaging of Pancreatic Ductal Adenocarcinoma. Clin. Cancer Res. Off. J. Am. Assoc. Cancer Res..

[19] Willmann J.K., Bonomo L., Carla Testa A., Rinaldi P., Rindi G., Valluru K.S., Petrone G., Martini M., Lutz A.M., Gambhir S.S. (2017). Ultrasound Molecular Imaging With BR55 in Patients with Breast and Ovarian Lesions: First-in-Human Results. J. Clin. Oncol.

[20] Kouijzer J.J.P., Lattwein K.R., Beekers I., Langeveld S.A.G., Leon-Grooters M., Strub J.-M., Oliva E., Mislin G.L.A., de Jong N., van der Steen A.F.W. (2021). Vancomycin-decorated microbubbles as a theranostic agent for Staphylococcus aureus biofilms. Int. J. Pharm..

[21] Wang X., Gkanatsas Y., Palasubramaniam J., Hohmann J.D., Chen Y.C., Lim B., Hagemeyer C.E., Peter K. (2016). Thrombus-Targeted Theranostic Microbubbles: A New Technology towards Concurrent Rapid Ultrasound Diagnosis and Bleeding-free Fibrinolytic Treatment of Thrombosis. Theranostics.

[22] Kwan J.J., Kaya M., Borden M.A., Dayton P.A. (2012). Theranostic oxygen delivery using ultrasound and microbubbles. Theranostics.

[23] Langeveld S.A.G., Meijlink B., Kooiman K. (2021). Phospholipid-coated targeted microbubbles for ultrasound molecular imaging and therapy. Curr. Opin. Chem. Biol..

[24] Smeenge M., Tranquart F., Mannaerts C.K., de Reijke T.M., van de Vijver M.J., Laguna M.P., Pochon S., de la Rosette J., Wijkstra H. (2017). First-in-Human Ultrasound Molecular Imaging With a VEGFR2-Specific Ultrasound Molecular Contrast Agent (BR55) in Prostate Cancer: A Safety and Feasibility Pilot Study. Investig. Radiol..

[25] Moccetti F., Brown E., Xie A., Packwood W., Qi Y., Ruggeri Z., Shentu W., Chen J., López J.A., Lindner J.R. (2018). Myocardial Infarction Produces Sustained Proinflammatory Endothelial Activation in Remote Arteries. J. Am. Coll Cardiol..

[26] Wang M., Hu R., Yang Y., Xiang L., Mu Y. (2019). In Vivo Ultrasound Molecular Imaging of SDF-1 Expression in a Swine Model of Acute Myocardial Infarction. Front. Pharmacol..

[27] Streeter J.E., Herrera-Loeza S.G., Neel N.F., Yeh J.J., Dayton P.A. (2013). A comparative evaluation of ultrasound molecular imaging, perfusion imaging, and volume measurements in evaluating response to therapy in patient-derived xenografts. Technol. Cancer Res. Treat..

[28] Turco S., El Kaffas A., Zhou J., Lutz A.M., Wijkstra H., Willmann J.K., Mischi M. (2019). Pharmacokinetic Modeling of Targeted Ultrasound Contrast Agents for Quantitative Assessment of Anti-Angiogenic Therapy: A Longitudinal Case-Control Study in Colon Cancer. Mol. Imaging Biol..

[29] Ingels A., Leguerney I., Cournède P.H., Irani J., Ferlicot S., Sébrié C., Benatsou B., Jourdain L., Pitre-Champagnat S., Patard J.J. (2020). Ultrasound Molecular Imaging of Renal Cell Carcinoma: VEGFR targeted therapy monitored with VEGFR1 and FSHR targeted microbubbles. Sci. Rep..

[30] Kosareva A., Abou-Elkacem L., Chowdhury S., Lindner J.R., Kaufmann B.A. (2020). Seeing the Invisible-Ultrasound Molecular Imaging. Ultrasound Med. Biol..

[31] Steinl D.C., Xu L., Ochoa-Espinosa A., Punjabi M., Kaufmann B.A. (2019). Non-invasive contrast enhanced ultrasound molecular imaging of inflammation in autoimmune myocarditis for prediction of left ventricular fibrosis and remodeling. PLoS ONE.

[32] Herbst E.B., Unnikrishnan S., Klibanov A.L., Mauldin F.W., Hossack J.A. (2019). Validation of Normalized Singular Spectrum Area as a Classifier for Molecularly Targeted Microbubble Adherence. Ultrasound Med. Biol.

[33] Woudstra L., Meinster E., Haren L.V., Kay A.M., Koopman M., Belien J.A.M., Morrison M.C., Rossum A.C.V., Helder M.N., Juffermans L.J.M. (2018). StemBell therapy stabilizes atherosclerotic plaques after myocardial infarction. Cytotherapy.

[34] Langeveld S.A.G., Schwieger C., Beekers I., Blaffert J., van Rooij T., Blume A., Kooiman K. (2020). Ligand Distribution and Lipid Phase Behavior in Phospholipid-Coated Microbubbles and Monolayers. Langmuir.

[35] Su J., Wang J., Luo J., Li H. (2019). Ultrasound-mediated destruction of vascular endothelial growth factor (VEGF) targeted and paclitaxel loaded microbubbles for inhibition of human breast cancer cell MCF-7 proliferation. Mol. Cell Probes.

[36] He Y., Zhang Y., Qin H.Y., Gu D.Y., Lu X., Hu J.X., Ye W.L., He G.B. (2020). Inhibitory effect of 5-FU loaded ultrasound microbubbles on tumor growth and angiogenesis. Bioorganic. Med. Chem. Lett..

[37] Wang Y., Li X., Liu L., Liu B., Wang F., Chen C. (2020). Tissue Targeting and Ultrasound-Targeted Microbubble Destruction Delivery of Plasmid DNA and Transfection In Vitro. Cell Mol. Bioeng..

[38] Ilovitsh T., Feng Y., Foiret J., Kheirolomoom A., Zhang H., Ingham E.S., Ilovitsh A., Tumbale S.K., Fite B.Z., Wu B. (2020). Low-frequency ultrasound-mediated cytokine transfection enhances T cell recruitment at local and distant tumor sites. Proc. Natl. Acad. Sci. USA.

[39] Klibanov A.L., Rasche P.T., Hughes M.S., Wojdyla J.K., Galen K.P., Wible J.H., Brandenburger G.H. (2004). Detection of Individual Microbubbles of Ultrasound Contrast Agents. Investig. Radiol..

[40] Skachkov I., Luan Y., van der Steen A.F., de Jong N., Kooiman K. (2014). Targeted microbubble mediated sonoporation of endothelial cells in vivo. IEEE Trans. Ultrason. Ferroelectr. Freq. Control.

[41] Kooiman K., Foppen-Harteveld M., van der Steen A.F., de Jong N. (2011). Sonoporation of endothelial cells by vibrating targeted microbubbles. J. Control. Release.

[42] Meijlink B., Skachkov I., van der Steen A.F.W., de Jong N., Kooiman K. (2021). The Preparation of Chicken Ex Ovo Embryos and Chorioallantoic Membrane Vessels as In Vivo Model for Contrast-Enhanced Ultrasound Imaging and Microbubble-Mediated Drug Delivery Studies. JoVE.

[43] Beekers I., Lattwein K.R., Kouijzer J.J.P., Langeveld S.A.G., Vegter M., Beurskens R., Mastik F., Verduyn Lunel R., Verver E., van der Steen A.F.W. (2019). Combined Confocal Microscope and Brandaris 128 Ultra-High-Speed Camera. Ultrasound Med. Biol..

[44] Feshitan J.A., Chen C.C., Kwan J.J., Borden M.A. (2009). Microbubble size isolation by differential centrifugation. J. Colloid Interface Sci..

[45] Papaioannou T., Stefanadis C. (2005). Vascular Wall Shear Stress: Basic Principles and Methods. Hell. J. Cardiol..

[46] Brooks Peter C., Clark Richard A.F., Cheresh David A. (1994). Requirement of Vascular Integrin αvβ3 for Angiogenesis. Science.

[47] Jenkins W.S., Vesey A.T., Vickers A., Neale A., Moles C., Connell M., Joshi N.V., Lucatelli C., Fletcher A.M., Spratt J.C. (2019). In vivo alpha-V beta-3 integrin expression in human aortic atherosclerosis. Heart.

[48] Pathak V., Nolte T., Rama E., Rix A., Dadfar S.M., Paefgen V., Banala S., Buhl E.M., Weiler M., Schulz V. (2021). Molecular magnetic resonance imaging of Alpha-v-Beta-3 integrin expression in tumors with ultrasound microbubbles. Biomaterials.

[49] Cao Y., Arbiser J., D’Amato R.J., D’Amore P.A., Ingber D.E., Kerbel R., Klagsbrun M., Lim S., Moses M.A., Zetter B. (2011). Forty-Year Journey of Angiogenesis Translational Research. Sci. Transl. Med..

[50] Tarbell J.M., Shi Z.-D. (2013). Effect of the glycocalyx layer on transmission of interstitial flow shear stress to embedded cells. Biomech. Model. Mechanobiol..

[51] Pedersen J.A., Boschetti F., Swartz M.A. (2007). Effects of extracellular fiber architecture on cell membrane shear stress in a 3D fibrous matrix. J. Biomech..

[52] Park J., Fan Z., Deng C. (2011). Effects of shear stress cultivation on cell membrane disruption and intracellular calcium concentration in sonoporation of endothelial cells. J. Biomech..

[53] Jalali S., del Pozo M.A., Chen K., Miao H., Li Y., Schwartz M.A., Shyy J.Y., Chien S. (2001). Integrin-mediated mechanotransduction requires its dynamic interaction with specific extracellular matrix (ECM) ligands. Proc. Natl. Acad. Sci. USA.

[54] Tzima E., del Pozo M.A., Shattil S.J., Chien S., Schwartz M.A. (2001). Activation of integrins in endothelial cells by fluid shear stress mediates Rho-dependent cytoskeletal alignment. EMBO J..

[55] Gupta I., Eisenbrey J., Stanczak M., Sridharan A., Dave J.K., Liu J.B., Hazard C., Wang X., Wang P., Li H. (2017). Effect of Pulse Shaping on Subharmonic Aided Pressure Estimation In Vitro and In Vivo. J. Ultrasound Med. Off. J. Am. Inst. Ultrasound Med..

[56] Lum J.S., Stobbe D.M., Borden M.A., Murray T.W. (2018). Photoacoustic technique to measure temperature effects on microbubble viscoelastic properties. Appl. Phys. Lett..

[57] Shekhar H., Smith N.J., Raymond J.L., Holland C.K. (2018). Effect of Temperature on the Size Distribution, Shell Properties, and Stability of Definity(R). Ultrasound Med. Biol..

[58] Sun C., Panagakou I., Sboros V., Butler M.B., Kenwright D., Thomson A.J., Moran C.M. (2016). Influence of temperature, needle gauge and injection rate on the size distribution, concentration and acoustic responses of ultrasound contrast agents at high frequency. Ultrasonics.

[59] Mulvana H., Stride E., Hajnal J.V., Eckersley R.J. (2010). Temperature dependent behavior of ultrasound contrast agents. Ultrasound Med. Biol..

[60] Guiot C., Pastore G., Napoleone M., Gabriele P., Trotta M., Cavalli R. (2006). Thermal response of contrast agent microbubbles: Preliminary results from physico-chemical and US-imaging characterization. Ultrasonics.

[61] Wang W., Liu G.J., Xie X.Y., Xu Z.F., Chen L.D., Huang G.L., Zhou L.Y., Lu M.D. (2012). Development and evaluation of lipid microbubbles targeted to alpha(v)beta(3)-integrin via biotin-avidin bridge. J. Microencapsul..

[62] Takalkar A.M., Klibanov A.L., Rychak J.J., Lindner J.R., Ley K. (2004). Binding and detachment dynamics of microbubbles targeted to P-selectin under controlled shear flow. J. Control. Release.

[63] Yang H., Xiong X.Y., Zhang L., Wu C.H., Liu Y.Y. (2011). Adhesion of bio-functionalized ultrasound microbubbles to endothelial cells by targeting to vascular cell adhesion molecule-1 under shear flow. Int. J. Nanomed..

[64] Weller G.E.R., Villanueva F.S., Klibanov A.L., Wagner W.R. (2002). Modulating targeted adhesion of an ultrasound contrast agent to dysfunctional endothelium. Ann. Biomed. Eng..

[65] Maul T., Dudgeon D., Beste M., Hammer D., Lazo J., Villanueva F., Wagner W. (2010). Optimization of Ultrasound Contrast Agents with Computational Models to Improve Selection of Ligands and Binding Strength. Biotechnol. Bioeng..

[66] Charoenphol P., Huang R.B., Eniola-Adefeso O. (2010). Potential role of size and hemodynamics in the efficacy of vascular-targeted spherical drug carriers. Biomaterials.

[67] Dayton P., Klibanov A., Brandenburger G., Ferrara K. (1999). Acoustic radiation force in vivo: A mechanism to assist targeting of microbubbles. Ultrasound Med. Biol..

[68] Schneider M., Broillet A., Tardy I., Pochon S., Bussat P., Bettinger T., Helbert A., Costa M., Tranquart F. (2012). Use of intravital microscopy to study the microvascular behavior of microbubble-based ultrasound contrast agents. Microcirculation.

[69] Serda R.E., Gu J., Bhavane R.C., Liu X., Chiappini C., Decuzzi P., Ferrari M. (2009). The association of silicon microparticles with endothelial cells in drug delivery to the vasculature. Biomaterials.

[70] Lindner J.R., Dayton P.A., Coggins M.P., Ley K., Song J., Ferrara K., Kaul S. (2000). Noninvasive Imaging of Inflammation by Ultrasound Detection of Phagocytosed Microbubbles. Circulation.

