# Supplementary material for: Theranostic Microbubbles with Homogeneous Ligand Distribution for Higher Binding Efficacy
Source: Pharmaceutics. 2022 Jan 28;14(2):311. doi: 10.3390/pharmaceutics14020311 (PMC8878664; doi:10.3390/pharmaceutics14020311)
Supplement: Supplementary file 1 [file pharmaceutics-14-00311-s001.zip › pharmaceutics-1554729-supplementary.pdf]

Supplemental Information

Manuscript: Theranostic microbubbles with homogeneous ligand distribution for higher binding efficacy

Simone A.G. Langeveld[1], Bram Meijlink[1], Inés Beekers[1,2], Mark Olthof[1], Antonius F.W. van der Steen[1], Nico de Jong[1,3], Klazina Kooiman[1]

[1] Department of Biomedical Engineering, Thoraxcenter, Erasmus University Medical Center, Rotterdam, the Netherlands.

[2] Department of Health, ORTEC B.V., Zoetermeer, the Netherlands

[3] Laboratory of Medical Imaging, Department of Imaging Physics, Delft University of Technology, Delft, the Netherlands.

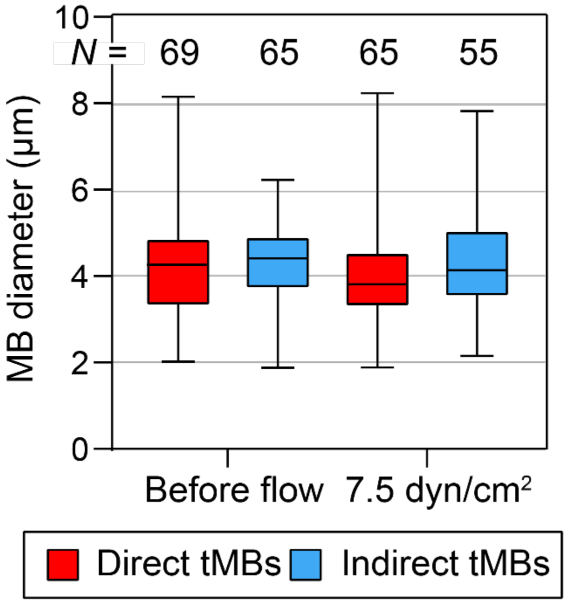

Figure S1. Diameter (µm) of bound direct and indirect targeted microbubbles (tMBs) from dissociation under flow assay with HUVEC monolayer *in vitro*. Diameters were measured in a frame from the recorded confocal time lapse video before flow was started, and at the end of the assay at 7.5 dyn/cm^2. Boxplots are shown with median, IQR, and whiskers from min to max.

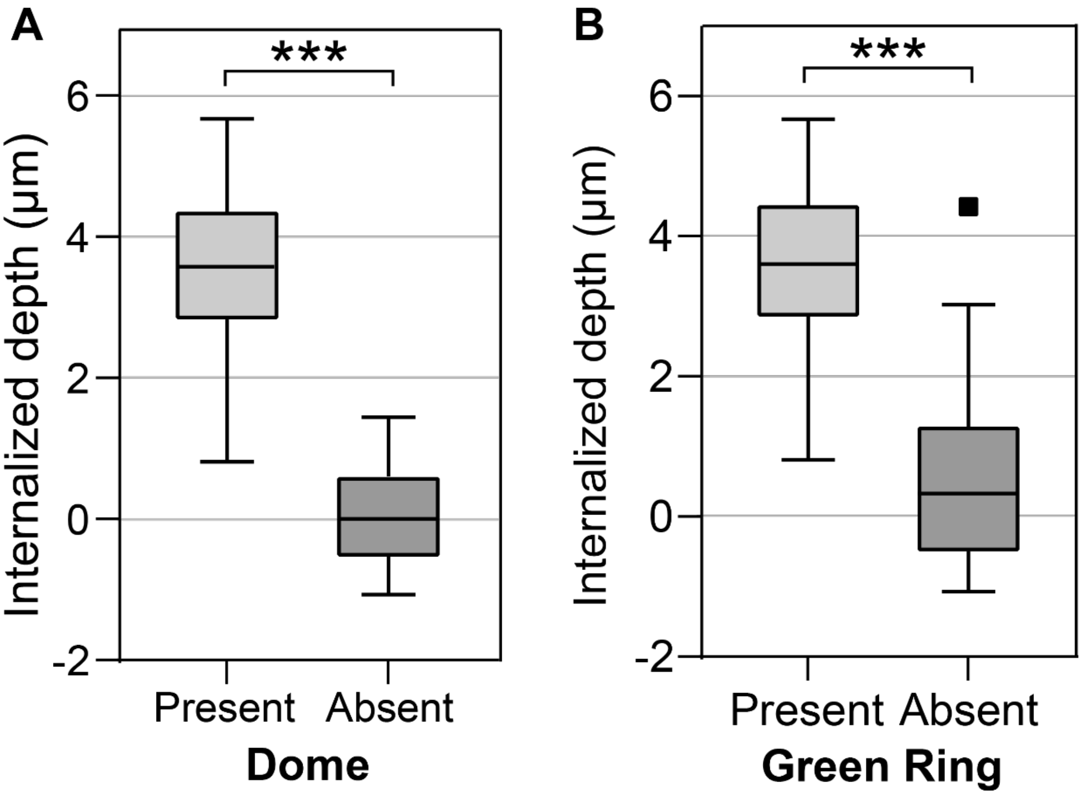

Figure S2. Internalized depth of (A) bound targeted microbubbles with a dome present (N = 41) or absent (N = 20), and (B) bound targeted microbubbles with a green ring present (N = 37) or absent (N = 24) in the CellMask Green channel, shown as boxplot with outlier in black (square). Significance is indicated with ***$p < 0.001$.

List of used abbreviations

BSA: bovine serum albumin; CAM: chorioallantoic membrane; DiD: 1,1'-dioctadecyl-3,3,3',3'-tetramethylindodicarbocyanine perchlorate; DiI: 1,1'-dioctadecyl-3,3,3',3'-tetramethylindocarbocyanine perchlorate; DSPC: 1,2-distearoyl-sn-glycero-3-phosphocholine; EGM: Endothelial Cell Growth Medium; FOV: field of view; HUVEC: human umbilical vein endothelial cell; ICAM: intracellular adhesion molecule; IQR: interquartile range; PBS: phosphate buffered saline; VCAM: vascular cell adhesion molecule; VEGFR2: vascular endothelial growth factor receptor 2
